# Discovery of substrate cycles in large scale metabolic networks using hierarchical modularity

**DOI:** 10.1186/s12918-015-0146-2

**Published:** 2015-02-13

**Authors:** Gautham Vivek Sridharan, Ehsan Ullah, Soha Hassoun, Kyongbum Lee

**Affiliations:** Department of Chemical and Biological Engineering, Tufts University, 4 Colby Street, Medford, MA 02155 USA; Department of Computer Science, Tufts University, 161 College Avenue, Medford, MA 02155 USA

**Keywords:** Substrate cycles, Modularity, Metabolic networks

## Abstract

**Background:**

A substrate cycle is a set of metabolic reactions, arranged in a loop, which does not result in net consumption or production of the metabolites. The cycle operates by transforming a cofactor, e.g. oxidizing a reducing equivalent. Substrate cycles have been found experimentally in many parts of metabolism; however, their physiological roles remain unclear. As genome-scale metabolic models become increasingly available, there is now the opportunity to comprehensively catalogue substrate cycles, and gain additional insight into this potentially important motif of metabolic networks.

**Results:**

We present a method to identify substrate cycles in the context of metabolic modules, which facilitates functional analysis. This method utilizes elementary flux mode (EFM) analysis to find potential substrate cycles in the form of cyclical EFMs, and combines this analysis with network partition based on retroactive (cyclical) interactions between reactions. In addition to providing functional context, partitioning the network into modules allowed exhaustive EFM calculations on smaller, tractable subnetworks that are enriched in metabolic cycles. Applied to a large-scale model of human liver metabolism (HepatoNet1), our method found not only well-known substrate cycles involving ATP hydrolysis, but also potentially novel substrate cycles involving the transformation of other cofactors. A key characteristic of the substrate cycles identified in this study is that the lengths are relatively short (2–13 reactions), comparable to many experimentally observed substrate cycles.

**Conclusions:**

EFM computation for large scale networks remains computationally intractable for exhaustive substrate cycle enumeration. Our algorithm utilizes a ‘divide and conquer’ strategy where EFM analysis is performed on systematically identified network modules that are designed to be enriched in cyclical interactions. We find that several substrate cycles uncovered using our approach are not identified when the network is partitioned in a more generic manner based solely on connectivity rather than cycles, demonstrating the value of targeting motif searches to sub-networks replete with a topological feature that resembles the desired motif itself.

**Electronic supplementary material:**

The online version of this article (doi:10.1186/s12918-015-0146-2) contains supplementary material, which is available to authorized users.

## Background

Cellular metabolism is exceedingly complex, involving the coordinated actions of many enzymes and regulatory molecules. A useful way to model cellular metabolism is to represent it as a network of biochemical reactions, where one enzyme-catalyzed reaction connects to another through shared reactants, products, and/or cofactors. A common theme in the study of these systems has been to relate a network’s function(s) to its layout or “topology”. For example, evolved networks typically harbor hubs to which less connected nodes attach as they join the network. This type of “small-world” property has been demonstrated for metabolic networks, with implications for evolution of metabolic functions. Another important property is modularity; i.e., these networks appear to contain smaller subsystems, analogous to integrated circuit modules comprising a larger digital circuit, which has practical implications for engineering biological cells to acquire new synthetic functions [1].

Several studies have shown that the modules in a metabolic network have hierarchy [2]. These studies utilized graphs, which facilitated the use of established algorithms to compute topological properties. Recently, we introduced a graph-based metric, termed Shortest Retroactive Distance (ShReD), to assess the degree of mutual influence, or retroactive interactions, between reactions in a metabolic network. This metric was used to hierarchically partition a metabolic network into modules that are enriched in allosteric feedback loops and metabolic cycles [3]. Partitioning a network to achieve enrichment of a particular feature, or motif, offers the benefit that the motif can be studied in its modular context.

One motif of particular interest is the substrate cycle, which refers to a set of reactions that forms a loop and does not lead to a net production or consumption of the participating metabolites. Thus defined, a substrate cycle would be thermodynamically infeasible without coupling the cycle’s operation to a thermodynamically favorable process such as ATP hydrolysis. Examples of experimentally confirmed substrate cycles involve opposing reactions in glycolysis and gluconeogenesis: inter-conversion of glucose and glucose 6-phosphate; fructose 6-phosphate and fructose 1,6-bisphosphate; and phosphoenolpyruvate and pyruvate. Often also labeled as futile cycles for the seemingly wasteful energy expenditure, substrate cycles have more recently been ascribed physiological functions, for example, thermogenesis [4]. Futile or substrate cycles are not to be confused with infeasible loops, which refer to thermodynamically infeasible cycles.

In the context of cellular homeostasis, substrate cycles could enable the cell to maintain an independent steady-state cycle flux, where the cycle flux can in theory fluctuate without directly altering other fluxes in the metabolic network, provided the cycle does not drastically disturb the cofactor pools. This feature could promote local robustness, which is a key property of modularity [5]. Conversely, if a substrate cycle significantly impacts the consumption or generation of a particular cofactor, then the enzymes of the cycle could be manipulated to *selectively* adjust the cofactor level while minimally perturbing other parts of metabolism. This type of direct and selective targeting of cofactors could be quite useful in modulating cellular energy expenditure. A substrate cycle in adipose tissue, esterification and hydrolysis of triglycerides, has been investigated as a target to treat obesity through manipulation of cellular bioenergetics [6]. Differential expression of substrate cycle enzymes has also been explored as a possible approach to modulate cancer cell metabolism [7].

In this light, comprehensively characterizing substrate cycles embedded throughout metabolism could not only shed light on the physiological role of this motif, but also discover potentially novel targets to manipulate metabolic function. Recently, Gebauer and coworkers showed that cyclical elementary flux modes (EFMs) are potential substrate cycles [8]. Using EFMEvolver [9], the authors found more than 200,000 cyclical EFMs, with a median length (defined as the number of reactions in an EFM) of 35. However, even this large number is likely an underestimate, as the authors focused on substrate cycles involving one specific cofactor, ATP. Given the very large number of potential substrate cycles, the analysis would be greatly facilitated by placing the cycles into context.

We present in this paper an approach for identifying substrate cycles in the context of hierarchical modularity. We employ the ShReD metric to partition a large-scale reconstruction of human liver metabolism (HepatoNet1) [10] into modules enriched in metabolic cycles, and conduct an EFM analysis *on each module* at varying levels of partition hierarchy. We find that it is possible to complete an exhaustive EFM enumeration for all but a small number of modules at the top of the hierarchy. Interestingly, many of the cyclical EFMs identified in this study span several metabolic modules, including transport, lipid synthesis, folate metabolism, sugar metabolism, and amino acid metabolism. The operation of these cyclical EFMs are coupled to many different cofactors as well as transport of inorganic hub compounds such as sulfate, phosphate, and hydronium ions.

## Methods

### Substrate cycle definition

An *elementary flux mode* (EFM) is a steady-state flux pattern in which flux proportions are fixed while their absolute magnitudes are indeterminate [11]. A sequence of reactions, or pathway, is an EFM if and only if it meets the following three conditions. First, the reactions along the pathway must proceed in a direction dictated by thermodynamic feasibility. Second, all metabolites internal to the network along the pathway are *balanced* under quasi steady-state conditions. That is, each internal metabolite does not accumulate or deplete. Third, each EFM must be independent from other EFMs in the network. In a *cyclical* EFM, the reactants of the first reaction in the pathway coincide with the products of the last reaction. By definition, a cyclical EFM contains a Strongly Connected Component (SCC), as each metabolite node in the cycle is reachable from any other metabolite node in the cycle. A substrate cycle is defined similarly as a cyclical EFM, except that not all internal metabolites are balanced, including cofactor metabolites. Here, we define cofactors as metabolites that contribute to the thermodynamic feasibility of a reaction, but do not participate as a recognizable reactant or product. Examples of cofactors include electron and phosphate group donors and receivers (e.g. NADH, NAD^+^, ATP, ADP, etc.) as well as inorganic molecules and ions involved in membrane transport. A *substrate cycle* balances reactant and product metabolites, but not the cofactor metabolites. Consequently, steady-state operation of a substrate cycle results in the net production or consumption of a cofactor. In the case of a network or sub-network that has been stripped of cofactors, a cyclical EFM represents a putative substrate cycle whose activity *in vivo* must be verified experimentally. Given the above definitions for cyclical EFM and substrate cycle, EFM enumeration techniques can be utilized to identify novel substrate cycles if the enumeration is performed on a network that does not include cofactors. The cyclical EFMs found in the modified network without the cofactors are not necessarily cyclical EFMs in the original network with the cofactors*.*

Due to the scalability limitation of existing EFM enumeration techniques, we pursued an approach that restricts EFM analysis to sub-networks or modules, rather than the entire network. While this approach has limitations for traditional EFM analysis [12] EFM enumeration is applied here to identify *only* cyclical EFMs. By definition, a cyclical EFM begins and ends with the same set of balanced metabolites. Therefore, all parts of a cyclical EFM found in a subnetwork reside in the subnetwork. Given that a sub-network is a subset of the larger parent network, it follows that a cyclical EFM found in a subnetwork will also be found in the larger parent network.

### Metabolic models and graph representation

We used a published model of human liver metabolism (HepatoNet1) [10] as the base network. From this base model, we derived two additional models, one for EFM analysis (hepEFM) and one for ShReD-based partitioning (hepShReD).

To derive hepEFM, we first removed reactions that either produce or consume an extracellular metabolite from the network, resulting in a graph comprising 1,418 reaction nodes. The rationale for removing reaction nodes connecting extracellular and intracellular metabolites was to focus on EFMs internal to the network. We then removed cofactors (for full list see Additional file 1: Removed_Metabolites), as these metabolites were not balanced for EFM computation to identify substrate cycles. The classification of a metabolite as either cofactor or main metabolite can in some cases be subjective. Even nucleotides with multiple phosphate groups (such as ATP), which are typically involved in many metabolic reactions as cofactors, sometimes participate in synthesis (or degradation) pathways as main reactants and products. For example, UTP(c) was not classified as a cofactor in this study, because it is a substrate for the production of UDP-N-acetylglucosamine(c) (r0115). Moreover, the degree connectivity of a metabolite does not provide a sufficiently rigorous criterion either; i.e. a highly connected “hub” metabolite is not necessarily a cofactor. Glutamine(c) is a hub metabolite that participates in more than 100 reactions (Additional file 1: Metabolite_Degrees); however, it is also a major substrate in numerous catabolic and anabolic reactions, e.g. peptide synthesis reactions, and was thus not classified as a cofactor in the present study. Conversely, we classified peroxisomal NADPH(p) and NADP(p) as cofactors, even though they participate in only 3 reactions and thus are not hub metabolites. These examples illustrate that the classification of metabolites as cofactors is context-dependent.

**Figure 1:**
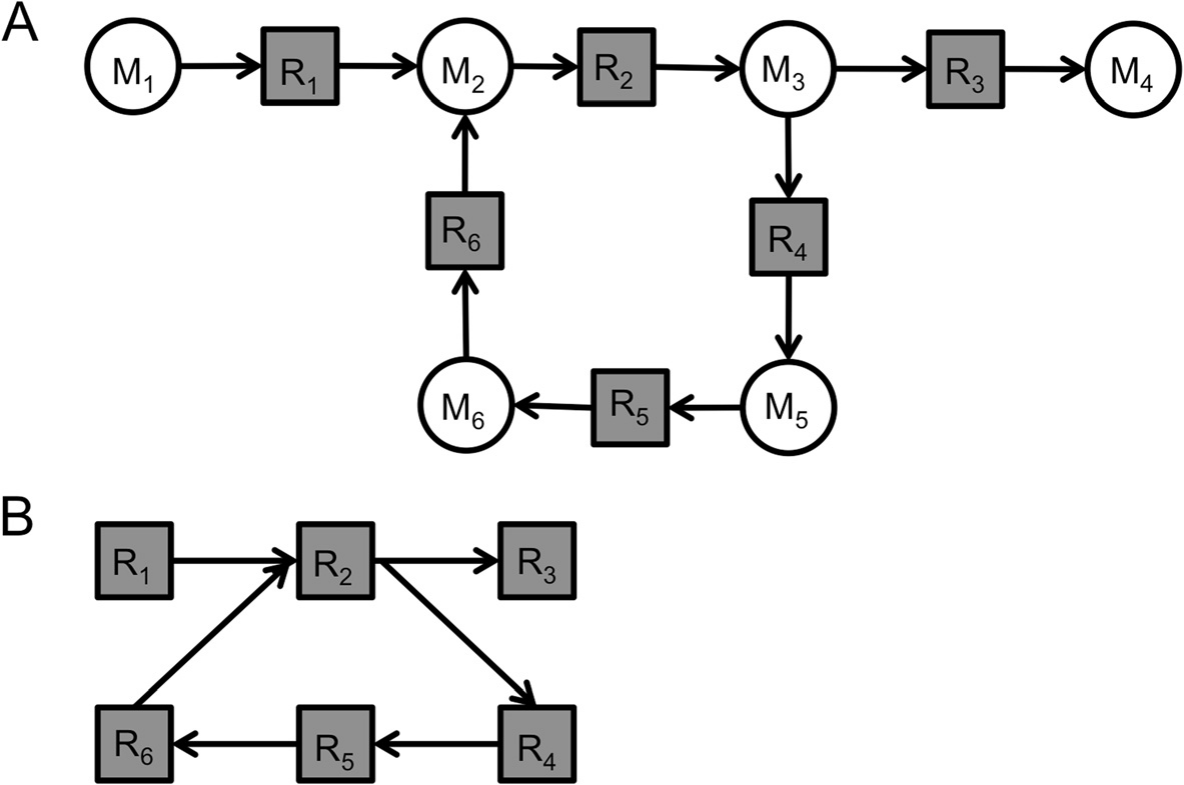
**Representation of metabolic networks as reaction-centric graphs for cyclical EFM analysis. (A)** An example of a bipartite graph representing a small metabolic network. Circles and square represent metabolites and reactions, respectively. A directed edge from a metabolite node to a reaction node indicates that the reaction consumes the metabolite. A directed edge from a reaction node to a metabolite node indicates that the reaction produces the metabolite. Once cofactors and dead-end metabolites (M1, M4) are removed, EFM analysis finds two flux modes: [R1, R2, R3] and [R2, R4, R5, R6]. The latter is a substrate cycle. **(B)** A reaction-centric graph of the network shown in **(A)**. Of the two elementary modes identified, only [R2, R4, R5, R6] comprises a SCC, and thus forms a cycle.

The hepShReD model for modularity analysis was also derived the same way as hepEFM except that certain cofactors such as ATP, NADH, and NADPH were retained in the model, since these molecules mediate important regulatory interactions between reactions that are captured using the ShReD metric (for cofactors retained in hepShReD, see Additional file 1: Removed_Metabolites). However, other inorganic cofactors (H_2_0, H^+^, sulfate, etc.) were still removed from hepShReD since reaction couplings determined based on the shared production and consumption of these metabolites are not meaningful in a metabolic context. The final hepShReD model was then abstracted as a reaction-centric directed graph based on the scheme illustrated in Figure 1. In addition, reactions producing or consuming extracellular metabolites were also removed, similar to the hepEFM model.

### Network partitioning

#### ShReD-based network partitioning

The hepShReD graph was partitioned into a hierarchical tree of modules based on the ShReD metric as described in our previous work [3]. Briefly, a ShReD value (length of the shortest directed cycle spanning two reaction nodes) was computed for every reaction pair in the parent network. The reaction pairs were ordered based on their corresponding ShReD values. The network was then partitioned into two subnetworks such that reaction pairs with larger ShReD values were split apart, whereas reaction pairs with shorter ShReD values were placed into the same sub-network. The ShReD-based partition was iterated on each successively formed subnetwork until the resulting partition no longer yielded a positive modularity score [13]. In case a partition produced a subnetwork that was not completely connected, the next iteration of the partition algorithm was performed on the connected components of this subnetwork. The ShReD-based partitioning algorithm takes into account both stoichiometric and regulatory (e.g. allosteric) interactions between reactions [3]. The latter could establish feedback loops that are important in determining retroactivity. However, the present study did not consider regulatory interactions, as the Hepatonet1 model did not include this information.

#### Newman-based network partitioning

The hepShReD graph was also partitioned using Newman’s metric [14], which only takes into account network connectivity. Briefly, Newman’s method seeks to place two nodes into the same module, if the number of edges between the nodes exceeds the number that is expected due to random connections. In our prior work, we found that the modules obtained using the ShReD method was more likely to contain retroactively connected pairs of nodes, i.e. cyclical pathways, compared to Newman’s method [3].

### Identifying substrate cycles as cyclical Elementary Flux Modes (EFMs) within modules

Each module in the hierarchical tree of partitions was analyzed for cyclical EFMs as follows. A stoichiometric matrix (S-matrix) was constructed for the reactions within a module by extracting the corresponding reaction columns from the S-matrix of hepEFM, and removing all dead-end metabolites without both a source and sink reaction. The module S-matrix was then analyzed using EFMTool [15] to enumerate all EFMs that can be formed using all or some subset of the reactions in the module. However, not every EFM necessarily represents a putative substrate cycle. For example, EFMtool would report both a cyclical and non-cyclical EFM for the network shown in Figure 1A. Thus, an additional check is needed. In a directed graph, every cycle forms a strongly connected component (SCC), as there is a path from each node to every other node. It follows that every nontrivial SCC contains at least one directed cycle. Based on this property, we can determine whether an EFM is cyclical by representing it as a directed graph, and checking whether all vertices in the corresponding graph belong to a single SCC. EFMtool automatically splits a reversible reaction *R* into two irreversible reactions *R*_*f*_ (forward) and *R*_*R*_ (reverse). However, the tool does not report EFMs involving only one reaction. Therefore, isomerization reactions at equilibrium (e.g. the inter-conversion of citrate and cis aconitate in the TCA cycle) would not be identified, even though they would be considered substrate cycles by our definition.

The enumeration of cyclical EFMs started at the terminal leaf modules of the hierarchical partition tree, and proceeded in order of increasing module height (defined as the maximum path length from the module to the root of the partition tree). The number of reactions in a module generally decreases from the root module (comprising all reactions in the model) with decreasing module height. Therefore, it was expected that the EFM calculations, which can be computationally intractable for large networks, would most likely complete within a reasonable run time (<1 h) for modules with small height. For a small number of modules at greater height (near the root module), EFMTool was indeed unable to complete the calculation in < 1 h. In this case, the EFM calculation moved on to another module at the same height. This process was repeated until no module remained that could be enumerated for EFMs in < 1 h. All computing was performed using a 2.83 GHz Intel Xeon E5440 CPU with 24 GB memory running Red Hat Linux.

### Net consumption of production of cofactors

Once a cyclical EFM was identified as a potential substrate cycle, the stoichiometric matrix of the original HepatoNet1 model was used to determine which cofactors were consumed or produced by this substrate cycle on a net basis. The stoichiometric matrix included all internal reactions of the HepatoNet1 model, including inter-compartmental (e.g. mitochondria-cytosol) exchange reactions.

## Results

### ShReD-based partition of HepatoNet1 model

**Figure 2:**
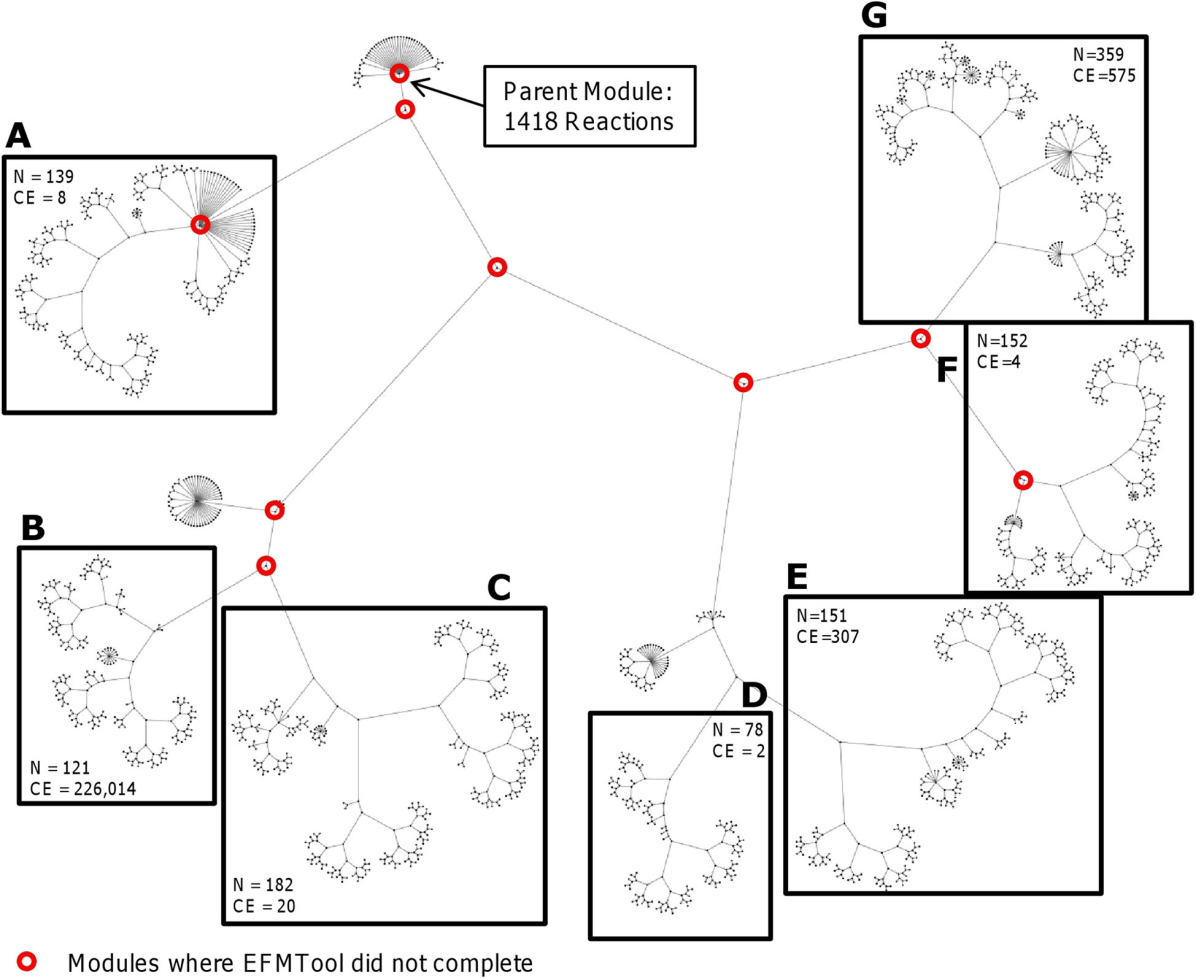
**The partition is visualized as a hierarchical graph where each node represents a module and edges emanating from a node represent a ShReD-based binary partition.** The partitioned network is divided into seven major groups, which are labeled **A-G**. Representative pathways and cyclical EFMs for each of these groups are reported in Table 1. N and CE, respectively, refer to the number of reactions and cyclical EFMs in the module.

Hierarchical partitioning of the hepShReD model yielded 2,098 modules, each of which comprised a subset of the original 1,418 intracellular reactions in the root module (see Additional file 1: ‘ShReD_Module_Partition’ for partition linkage by ID, and Sheet: ‘Modules’ for reaction composition of each module). Figure 2 illustrates the resulting partition as a graph. Each node represents a module, and each pair of edges emanating from that node represents a ShReD-based binary partition. The enumeration of EFMs completed for all but nine modules that are closest to the root node in the hierarchy. These nine modules contained too many reactions for EFMtool to complete the calculation in a reasonable amount of time. Interestingly, the number of cyclical EFMs in a module did not correlate with the size (number of reactions) of the module, and the density of cyclical EFMs (number of EFMs/reactions in module) varied greatly across the module hierarchy. For example, module # 144,976 (Figure 2A) contains 139 reactions and only 8 cyclical EFMs, whereas module # 143,829 (Figure 2B) contains 121 reactions and 226,014 cyclical EFMs (Table 1).

**Table 1:** **Representative modules and cyclical EFMs**

**Module ID**	**Metabolic function(s)**	**Representative cyclical EFM**	**Newman**
Figure 2A: #144976	Cholesterol synthesis	r1136: 4alpha-Methylzymosterol-4-carboxylate (r) + NADP+(r) ↔ 3-Keto-4-methylzymosterol(r) + NADPH(r).	Yes
	Lipoprotein synthesis	r1137: NAD+(r) + 4alpha-Methylzymosterol-4-carboxylate (r) ↔ NADH(r) + CO2(r) + 3-Keto-4-methylzymosterol(r)	
Figure 2B: #143830	TCA cycle	r0829: Succinate(c) + Sulfate(m) ↔ Succinate(m) + Sulfate(c)	No
	Mitochondria-cytosol exchange	r0831: Malate(c) + Pi(m) ↔ Malate(m) + Pi(c)	
		r0931: Isocitrate(m) + Malate(c) ↔Isocitrate(c) + Malate(m)	
		r0915: Citrate(c) + Succinate(m) ↔ Citrate(m) + Succinate(c)	
		r0917: Citrate(c) + Isocitrate(m) ↔ Citrate(m) + Isocitrate(c)	
Figure 2C: #143829	β-Oxidation	r0223: 2-Methyl-3-oxopropanoate(m) + CoA(m) + NAD+(m) → Propanoyl-CoA(m) + CO2(m) + NADH(m)	Yes
	Glutamate and proline metabolism	r0414: ATP(m) + Propanoyl-CoA(m) + HCO3-(m) → ADP(m) + Pi(m) + Methylmalonyl-CoA(m)	
	Ketone body synthesis	r0571: Methylmalonyl-CoA(m) + H2O(m) ↔ Methylmalonate(m) + CoA(m)	
	TCA Cycle	r0643: 2-Methyl-3-oxopropanoate(m) + NAD+(m) + H2O(m) ↔ Methylmalonate(m) + NADH(m)	
Figure 2D: #142886	NADH(c) metabolism	r0267: CMP-N-acetylneuraminate(c) + O2(c) + NADH(c) ↔ CMP-NeuNGc(c) + NAD+(c) + H2O(c)	No
		r0269: CTP(n) + N-Acetylneuraminate(n) ↔ PPi(n) + CMP-N-acetylneuraminate(n)	
		r0400: NAD+(c) + O2(c) + N-Acetylneuraminate(c) ↔ NeuNGc(c) + NADH(c) + H2O(c)	
		r0668: NeuNGc(c) + CTP(c) ↔ PPi(c) + CMP-NeuNGc(c)	
		r1461: CMP-N-acetylneuraminate(c) ↔ CMP-N-acetylneuraminate(n)	
		r1462: N-Acetylneuraminate(c) ↔ N-Acetylneuraminate(n)	
Figure 2E: #142887	Lipid biosynthesis	r0225: THF(c) + NADP+(c) ↔Dihydrofolate(c) + NADPH(c)	Yes
	Folate metabolism	r0227: 10-Formyl-THF(c) + H2O(c) + NADP+(c) → THF(c) + CO2(c) + NADPH(c)	
	NADPH(c) metabolism	r0293: 5,10-Methylene-THF(c) + NADP+(c) ↔ 5,10-Methenyl-THF(c) + NADPH(c)	
		r0371: 5,10-Methenyl-THF(c) + H2O(c) ↔ 10-Formyl-THF(c)	
		r0501: dUMP(c) + 5,10-Methylene-THF(c) ↔ Dihydrofolate(c) + dTMP(c)	
Figure 2F: #142411	Acyl-CoA activation in cytosol	r0066: ATP(c) + Acetate(c) + CoA(c) → AMP(c) + PPi(c) + Acetyl-CoA(c)	No
	Lipoprotein synthesis	r0485: Glucosamine-6P(c) + Acetyl-CoA(c) → CoA(c) + N-Acetylglucosamine-6P(c)	
		r0486: N-Acetylglucosamine-6P(c) + H2O(c) ↔ Glucosamine-6P(c) + Acetate(c)	
Figure 2G: #141811	Sugar metabolism	r0487: Fructose-1,6PP(c) + H2O(c) → Fructose-6P(c) + Pi(c)	No
	Amino acid metabolism	r0736: ATP(c) + Fructose-6P(c) → ADP(c) + Fructose-1,6PP(c)	
	Protein synthesis	r0129: GSH(c) + H2O(c) ↔ Glutamate(c) + Cys-Gly(c)	
		r0131: ATP(c) + gamma-Glutamyl-cysteine(c) + Glycine(c) → ADP(c) + Pi(c) + GSH(c)	
		r0212: ATP(c) + Glutamate(c) + Cysteine(c) → ADP(c) + Pi(c) + gamma-Glutamyl-cysteine(c)	
		r0214: H2O(c) + Cys-Gly(c) ↔ Cysteine(c) + Glycine(c)	

The first column lists the corresponding panel in Figure 2 and module ID (referenced in Additional file 1: ‘Modules’ for complete reaction list). The second column lists conventional textbook metabolic pathways/functions associated with reactions contained in the module. The third column provides a sample cyclical EFM identified for a given module. The fourth column indicates whether or not the cyclical EFM was identified when the network was partitioned using Newman’s connectivity-based modularity metric.

### Module functions and representative substrate cycles

For each boxed group of modules shown in Figure 2, Table 1 lists representative metabolic functions and cyclical EFMs. We are careful to use the term ‘cyclical EFM’, since not every cyclical EFM is necessarily an active substrate cycle *in vivo*. For example, when all reactions in a cyclical EFM are reversible, the flux direction predicted by the EFM calculation may be actually opposite of the actual flux direction in the cell. In our analysis of HepatoNet1, we identified a large number of cyclical EFMs involving two reversible reactions that carry out the same biochemical transformation using different cofactors. An illustrative example of this motif is found in module # 144,976 (Figure 2A). This module includes reactions in cholesterol synthesis and very low-density lipoprotein (VLDL) metabolism. The conversion of 3-keto-4-methylzymosterol(r) to 4alpha-methylzymosterol-4-carboxylate(r) is mediated by NADPH/NADP^+^ in one reaction and NADH/NAD^+^ in the other. Substrate cycling in this case could exchange NADPH into NADH or NADH into NADPH, depending on the direction. This substrate cycle could be active, if the NADPH/NADP^+^ ratio is sufficiently different from the NADH/NAD^+^ ratio, allowing for both the forward and reverse reactions to be thermodynamically feasible.

**Figure 3:**
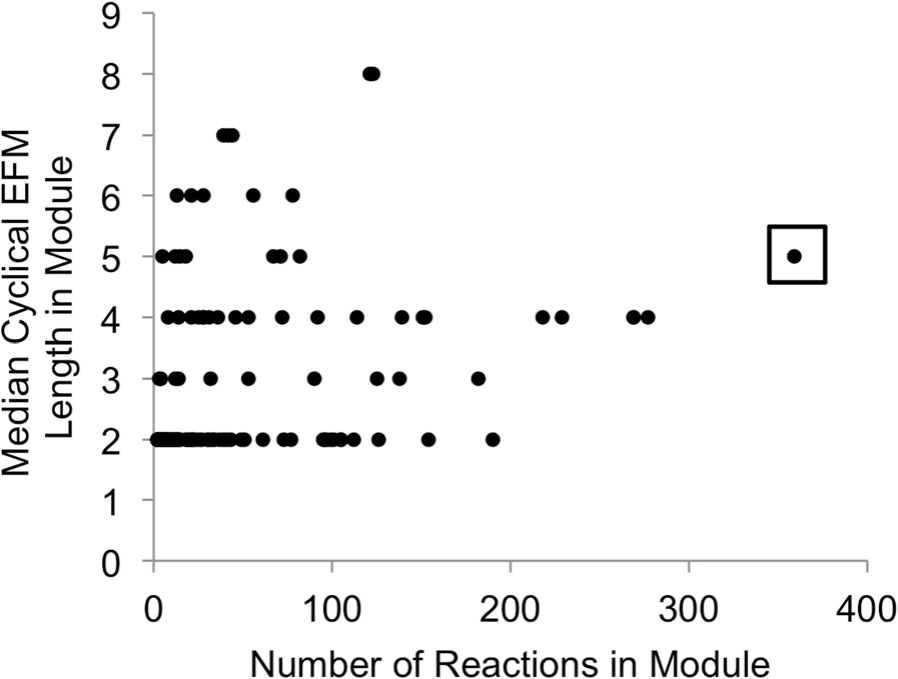
**For each module, the median cyclical EFM length is plotted against the number of reactions in the module.** The median cyclical EFM lengths for the Hepatonet1 modules span between 2 and 8 reaction steps.

Another trend is that many cyclical EFMs involve transport reactions across membranes. In particular, 221,576 of the 226,014 cyclical EFMs (>98%) found in module # 143,830 (Figure 2B) include at least one reaction involved in the transport of TCA cycle intermediates such as citrate, malate, and succinate across the mitochondrial membrane (Additional file 1: TCA_Transport_Reactions). Depending on the cycling direction, the function of such substrate cycles could be to transfer protons or phosphate ions across the mitochondrial membrane without bringing about a net change in other metabolite concentrations across the membrane. These cyclical EFMs are part of a module that also contains several TCA cycle carbon backbone reactions, underscoring the coupling between energy production reactions and membrane charge transfer reactions required to mediate the proton gradient for oxidative phosphorylation.

In some cases, the placement of cyclical EFMs into a particular module is intuitive and consistent with experimental observations. For example, module # 141,811 (Figure 2G) comprises reactions in sugar metabolism, amino acid metabolism, and protein synthesis. Within this module, the cyclical EFM involving the inter-conversion of fructose-6-phosphate and fructose-1,6-bisphosphate is part of both glycolysis and gluconeogenesis. Similarly, the cyclical EFM involving the production and degradation of glutathione at the expense of ATP directly influences the metabolism of glutamate, cysteine, and glycine, the three constituent amino acids of glutathione.

We also found less obvious associations between cyclical EFMs and ShReD-based modules. The latter tend to group together reactions that span several distinct textbook-defined pathways based on the shared production and consumption of metabolic cofactors [3]. For example, a cyclical EFM in module # 142,886 (Figure 2D) that involves N-glycolyneuraminate (NeuNGc) metabolism belongs to the same module as lactate dehydrogenase, an enzyme in glycolysis. Our analysis predicts a close interaction between these reactions that have ostensibly unrelated functions, because they share production and consumption of the same cofactor (NADH) in the same cellular compartment (cytosol). Similarly, a cyclical EFM in module # 142,887 (Figure 2E) represents the one-carbon cycle in folate metabolism that results in net production of NADPH, which directly connects to lipid biosynthesis and other reactions that require this cofactor.

To assess the effect of the partitioning method on the identification of substrate cycles, we compared the results obtained on modules generated using the ShReD metric against results obtained using Newman’s metric, which takes into account network connectivity but is not specifically designed to favor modules that are enriched with cyclical interactions. Overall, we found that the modules generated using Newman’s metric did not contain many of the substrate cycles identified using ShReD-based modules. In particular, 4 of the 7 representative substrate cycles listed in Table 1 are not identified when the cyclical EFM search was performed on Newman-based modules (see column 4 in Table 1). It should be noted, however, that a module-by-module comparison could not be performed, as the two metrics yielded different modules of varying sizes. In this regard, the partition metric clearly impacts the identification of substrate cycles in modules.

### Cofactors involved in substrate cycles

An important motivation for this work is to comprehensively identify the full range of cofactors that are consumed or produced by potential substrate cycles. Table 2 lists 30 cofactors that are the most frequently metabolized by cyclical EFMs. The complete list is provided in Additional file 1: ‘TableS2_Complete’. The vast majority of cyclical EFMs are associated with cytosolic hydronium, phosphate, and sulfate ions, suggesting a large number of substrate cycles involved in membrane transport (Module 143,830, Figure 2B). For these cyclical EFMs, the net consumption in one compartment is balanced by the net production in the other compartment and vice versa, which is reflected by the similar number of EFMs involved with both the cytosolic and mitochondrial pool of an ion metabolite (Table 2). Interestingly, the cofactor pairs NADH(c)/NAD^+^(c) and NADPH(c)/NADP^+^(c) are metabolized by similar numbers of cyclic EFMs. According to HepatoNet1’s annotation, many reversible reactions can be catalyzed by either cofactor. Consequently, our algorithm identifies cycles where one reaction consumes NADH(c) in one direction and the other reaction consumes NADPH(c) in the reverse direction. Lastly, we found ~700 cyclical EFMs that utilize various deoxynucleotide triphosphates, suggesting substrate cycles may be involved in nucleic acid metabolism.

**Table 2:** **Number of cyclical EFMs associated with each cofactor**

**Cofactor**	**Number of cyclical EFMs: net consumed**	**Number of cyclical EFMs: net produced**
H+(c)	70504	70446
H+(m)	70446	70504
Pi(c)	55051	55102
Pi(m)	55051	55055
Sulfate(c)	33046	33046
Sulfate(m)	33046	33046
Sulfite(c)	33046	33046
Sulfite(m)	33046	33046
H_2_O(c)	15015	14960
H_2_O(m)	14958	14956
ATP(c)	155	217
ADP(c)	208	152
dATP(c)	143	120
UDP(c)	106	151
dUTP(c)	155	96
CTP(c)	94	47
CDP(c)	57	82
GDP(c)	59	59
dGTP(c)	58	58
GTP(c)	69	45
dGDP(c)	44	68
dAMP(c)	27	37
dUDP(c)	25	37
dCTP(c)	23	35
dCDP(c)	23	35
NAD^+^(c)	24	24
NADH(c)	24	24
NADPH(c)	19	20
NADP^+^(c)	20	19
PPi (c)	3	24

For each of the top 30 cofactors that most frequently participate in a cyclical EFM (column 1), the table reports the number of cyclical EFMs that consume (column 2) or produce (column 3) the cofactor. The cofactors are sorted in descending order based on the total number of cyclical EFMs in which they participate. The letter in the parentheses indicates the cellular compartment for each cofactor as either cytosolic (c) or mitochondrial (m).

One consequence of our modular approach is that the resultant cyclical EFMs are contained within a module, limiting the size of substrate cycles that can be discovered. Previously, Gebauer and coworkers reported a median cyclical EFM length of 35 reactions for a genome-scale human metabolic network, with some cycles spanning up to 100 reactions. In this study, the longest cyclical EFM spanned 13 reactions. The median length of a cyclical EFM ranges from 2 to 8, depending on the module. We did not find a significant correlation between mean cyclical EFM length and module size, suggesting that larger modules may not necessarily possess longer cyclical EFMs (Figure 3). For module # 141,811 (boxed in Figure 3), which is the largest module analyzed for EFMs in this study, the distribution of cyclical EFM lengths is bi-modal (Additional file 1: Figure S1). Multi-model distribution of cyclical EFM lengths was also reported previously [8].

## Discussion

In this study, we utilized a network partition approach to identify substrate cycles in the context of metabolic modules. Substrate cycles have been found experimentally in many parts of metabolism, particularly across opposing reactions of glycolysis and gluconeogenesis. However, due to the complexity of metabolic networks, comprehensive characterization has been difficult. In conjunction with large-scale metabolic models, computational approaches offer the benefit that they can systematically consider most (if not all) reactions in a cell type or organism.

Performing motif searches on large-scale networks is challenging, and often intractable, unless the motif is very small. One way to address this problem is to place a upper limit on the number of components in the motif, but this can be arbitrary [16]. On the other hand, finding substrate cycles by enumerating all directed cycles of any size is computationally intractable, because the number of cycles may grow non-linearly with the number of reactions in a cycle. Recently, Gebauer and coworkers addressed this challenge by narrowing their search to cyclical EFMs that metabolize a specific cofactor, ATP. While the same algorithm could be used to target other cofactors, there is the potential drawback that relationships between cyclical EFMs involving different cofactors could be missed. In our work, we instead apply a constraint on the search space. To identify appropriate search spaces while retaining contextual information, we hierarchically partition the network into modules such that the modules are enriched in the desired motif, i.e. metabolic cycles. The trade-off here is that we compromise on the possibility of finding longer cyclical EFMs, as shown by the relatively short cyclical EFM lengths (Figure 3). Even for the module with the largest number of cyclical EFMs, the lengths range between 2 and 13 reactions (Additional file 1: Figure S1), compared to lengths of up to 100 reactions that can be identified from a global analysis [8]. The benefit of a modular approach is that one can be exhaustive in searching for all cyclical EFMs in a given module at a particular hierarchical resolution, which can then facilitate the association of a cyclical EFM with a recognizable metabolic function. Defining an appropriate neighborhood in the network to search for a motif can be challenging, and has often relied on a heuristic such as setting a fixed path distance around a reaction of interest. ShReD-based partition provides a systematically derived grouping of reactions that are enriched with metabolic cycles, and thus defines a natural search space for substrate cycles.

In this paper, we have been thus far careful not to use the terms ‘cyclical EFM’ and ‘substrate cycle’ interchangeably. The cyclical EFMs were identified solely based on the stoichiometry and reaction directionality as specified in the published model. While these EFMs could represent substrate cycles, it cannot be concluded that the substrate cycle is active *in vivo*. For example, many of the cyclical EFMs involve two reversible reactions where one consumes NADP^+^ and produces NADPH in the forward direction, and the other consumes NADH and produces NAD^+^ in the reverse direction (Table 1, module # 144,976, Figure 2A). However, whether or not these reactions can indeed operate in opposite directions at the same time with a net conversion of NADP to NADPH at the expense of NADH (or vice versa) depends on the intracellular conditions (e.g. reactant and product concentrations) influencing the thermodynamic driving force. One example of this type of substrate cycle that has been observed *in vivo* occurs between isocitrate and ketoglutarate, where NAD-dependent isocitrate dehydrogenase (IDH) catalyzes the conversion of isocitrate to ketoglutarate and NADP-dependent IDH catalyzes the conversion of ketoglutarate back to isocitrate [17].

The thermodynamic feasibility of these substrate cycles could in principle be determined based on Gibbs free energy estimates and experimental flux data [18]. Several earlier studies have considered thermodynamic constraints specifically in the context of cycles. Beard and co-workers formulated the “loop law”, which states that the net flux around a loop internal to a reaction network must be zero [19]. It has been shown that these internal loops could be identified using extreme pathway analysis [20] or by enumerating linear combinations of vectors that span the null space of the underlying reaction network. While mathematically rigorous, enumerating null space vector combinations can become intractable for large-scale problems, similar to EFM analysis. More recently, additional methods have been described that utilize the Gibbs free energy criterion of the Second Law [21] to eliminate thermodynamically infeasible reaction cycles. A computationally attractive alternative that does not rely on Gibbs energy parameters was described by Schellenberger and coworkers, who showed that the loop law can be integrated into constraint-based flux analysis by reformulating the optimization problem as a modified mixed integer program (MIP) [22]. The main difference between the present work and these previous studies lies in the type of loop reactions that are targeted for identification. The aforementioned studies sought to eliminate infeasible loops, i.e. futile cycles, when computing flux distributions, whereas the present work seeks to identify feasible loops, i.e. substrate cycles.

In interpreting the results, one also has to consider the “goodness” of the modular partitions. Our algorithm places reactions into groups so as to enrich the modules in cycles. However, it is possible that the algorithm finds a locally optimal result due to the nonlinearity of the problem. If this happens early in the partition process, then it is possible that our algorithm does not identify some substrate cycles. For example, Peterson and coworkers have reported on a metabolic cycle in the rat liver involving pyruvate carboxylase, malate dehydrogenase, and malic enzyme with net oxidation of NADH and NADPH [23]. Our algorithm does not identify this substrate cycle, because cytosolic malic enzyme groups with mitochondrial pyruvate carboxylase and malate dehydrogenase only near the parent module (# 141,802), which is too large for EFM enumeration. One way to address this limitation is to include activity data. Recently, we showed that weighting the edges with metabolic flux data results in different modularity, reflecting the metabolic state of the system [13]. We found that weighting the edges to reflect reaction engagements better ensured that highly active cycles are prioritized in partitioning the network. The practical challenge in adopting this scheme to analyze large-scale networks is obtaining flux data from isotope labeling experiments. On the other hand, it has been shown that methods such as flux balance analysis (FBA) and flux variability analysis (FVA) could reasonably circumscribe the feasible space of flux distributions for a number of genome-scale models.

Prospectively, the discovery of new substrate cycles could yield novel drug targets for metabolic diseases such as obesity, diabetes and cancer [24]. For example, a proposed mechanism for the insulin sensitizing action of thiazolidinedione (TZD) drugs is to induce glycerol kinase gene expression and thereby promote a substrate cycle between lipolysis and fatty acid esterification [25]. Induction of glycerol kinase and activation of the “futile” cycle could reduce the efflux of free fatty acids, whose levels in serum correlate with obesity-related insulin insensitivity. While it remains to be established that this futile cycle is a dominant mechanism *in vivo*, other studies have corroborated that the glycerol kinase-dependent substrate cycle is a viable mechanism for limiting free fatty acid efflux from human adipose tissue [26]. In addition, a study on cancer cachexia found that the activity of triglyceride-fatty acid substrate cycle was elevated in tumors of murine white adipose tissue due to differential expression of the cycle enzymes [27], which sets up an intriguing possibility of modulating tumor growth by manipulating these enzymes. The Cori cycle is another substrate cycle with increased activity in cachectic patients, where lactic acid produced in the tumor is converted to glucose in the liver at the expense of ATP [28].

The methodology presented in this paper could be extended to identify other substrate cycles that span multiple organ systems, similar to the Cory Cycle. This will obviously require metabolic models that describe more than one cell type or tissue. In this regard, genome-scale metabolic models representing the metabolic networks of multiple interacting cell types [29] or organisms [30] offer exciting possibilities to identify novel, inter-organ or inter-species substrate cycles in the context of recognizable biochemical functions as defined by the corresponding module.

## Conclusions

In this study, we present a novel algorithm for the discovery of substrate cycles in large scale metabolic networks by identifying cyclical EFMs in hierarchical modules designed to preserve cyclical interactions using our ShReD metric. Since ShReD-based modules naturally group reactions together based on the shared consumption and production of metabolic cofactors, they serve as a suitable search space to identify substrate cycles that have a net consumption or production of specific cofactors. We show that several representative substrate cycles are identified within their functional context based on known metabolic pathways whose reactions are present in each module. More importantly, we show that many of those substrate cycles are not identified if alternative partition metrics are used instead of ShReD to determine the hierarchical modularity, demonstrating the value of our ShReD-based algorithm for discovering substrate cycles.

The methodology used to identify the substrate cycle motif can be used to exhaustively enumerate cyclical EFMs in hierarchically arranged modules, allowing the analysis to circumvent the scalability limitation of existing EFM analysis tools. Compared to connectivity-based metrics, the ShReD metric used here is more likely to preserve cyclical interactions among network components, and thus is well suited to generating hierarchically partitioned modules for cyclical EFM analysis. To illustrate the advantage of ShReD-based partitioning, we show that several substrate cycles identified in the ShReD modules and reported in the literature are not identified in modules generated using a representative connectivity-based partition metric.

## Additional file

Additional file 1:
**Additional tables referenced in the text as well as the reaction composition of each module identified using ShReD-based partitioning of the Hepatonet1 liver network.**
